# Ataluren suppresses a premature termination codon in an MPS I-H mouse

**DOI:** 10.1007/s00109-022-02232-0

**Published:** 2022-07-20

**Authors:** Dan Wang, Xiaojiao Xue, Gwen Gunn, Ming Du, Amna Siddiqui, Marla Weetall, Kim M. Keeling

**Affiliations:** 1 Department of Biochemistry and Molecular Genetics, University of Alabama at Birmingham, BBRB 444, 1720 Second Avenue South, Birmingham, AL 35294-2170 USA; 2 Horae Gene Therapy Center and RNA Therapeutics Institute, University of Massachusetts Chan Medical School, Worcester, MA 01605 USA; 3 PTC Therapeutics Incorporated, South Plainfield, NJ 07080 USA; 4 Department of Human Genetics, Emory University, Atlanta, GA 30322 USA

**Keywords:** Nonsense mutation, PTC suppression, Readthrough, Ataluren, Mucopolysaccharidosis I-Hurler

## Abstract

**Abstarct:**

Suppressing translation termination at premature termination codons (PTCs), termed readthrough, is a potential therapy for genetic diseases caused by nonsense mutations. Ataluren is a compound that has shown promise for clinical use as a readthrough agent. However, some reports suggest that ataluren is ineffective at suppressing PTCs. To further evaluate the effectiveness of ataluren as a readthrough agent, we examined its ability to suppress PTCs in a variety of previously untested models. Using NanoLuc readthrough reporters expressed in two different cell types, we found that ataluren stimulated a significant level of readthrough. We also explored the ability of ataluren to suppress a nonsense mutation associated with Mucopolysaccharidosis I-Hurler (MPS I-H), a genetic disease that is caused by a deficiency of α-L-iduronidase that leads to lysosomal accumulation of glycosaminoglycans (GAGs). Using mouse embryonic fibroblasts (MEFs) derived from *Idua-W402X* mice, we found that ataluren partially rescued α-L-iduronidase function and significantly reduced GAG accumulation relative to controls. Two-week oral administration of ataluren to *Idua-W402X* mice led to significant GAG reductions in most tissues compared to controls. Together, these data reveal important details concerning the efficiency of ataluren as a readthrough agent and the mechanisms that govern its ability to suppress PTCs.

**Key messages:**

- Ataluren promotes readthrough of PTCs in a wide variety of contexts.
- Ataluren reduces glycosaminoglyan storage in MPS I-H cell and mouse models.
- Ataluren has a bell-shaped dose–response curve and a narrow effective range.

## Introduction

Nonsense suppression is currently being explored as a potential therapeutic approach for genetic diseases caused by in-frame premature termination codons (PTCs), commonly referred to as nonsense mutations [1]. PTCs terminate translation of an mRNA prior to the production of a full-length protein, resulting in the generation of a truncated polypeptide that often lacks normal function and/or is unstable. Translation termination, which is mediated by a termination complex minimally composed of eRF1 and eRF3 [2], is normally a very efficient process. However, aminoacyl tRNAs that base pair with two of the three nucleotides of a termination codon, termed near-cognate aminoacyl tRNAs, naturally compete with the termination complex for PTC binding at low levels [3]. If a near-cognate aminoacyl tRNA becomes accommodated into the ribosomal acceptor site, its associated amino acid can be incorporated into the nascent polypeptide at the site of the PTC. This so-called “readthrough” mechanism allows translation elongation to continue in the correct ribosomal reading frame, producing a full-length protein that is likely to possess at least partial protein function.

Several low molecular weight compounds have been identified that enhance the suppression of translation termination at PTCs [4]. Aminoglycosides are the best-characterized readthrough agents. A subset of this class of antibiotic has been shown to effectively suppress PTCs and rescue deficient protein function in multiple cell- and animal-based genetic disease models and also in clinical trials [5]. However, traditional aminoglycosides are generally precluded from long-term clinical use due to their potential to induce ototoxicity [6] and nephrotoxicity [7, 8]. Importantly, the mechanisms behind aminoglycoside-mediated toxicity appear to be largely unrelated to their role in suppressing termination at cytoplasmic ribosomes [9–13]. This prompted a more extensive search for safe, effective readthrough agents. Ataluren (Translarna™; PTC124) was identified from a high-throughput screen as a compound that efficiently suppresses PTCs in mammalian cells without toxicity [14]. Ataluren was subsequently found to be safe for human use [15, 16].

While numerous investigations have shown ataluren has the ability to suppress a variety of disease-associated PTCs using in vitro and in vivo models [5, 17] and in clinical trials [5], the negative results of other studies have led to skepticism concerning the ability of ataluren to effectively suppress PTCs [18, 19]. In this study, we investigated whether we could determine potential reasons for the divergent results that have been reported for the effectiveness of ataluren as a readthrough compound.

To further explore the ability of ataluren to suppress PTCs, we used a series of novel NanoLuc-based readthrough reporters. We found that ataluren was more effective at suppressing NanoLuc PTCs than the clinically relevant readthrough compounds, gentamicin and amlexanox. We also examined the ability of ataluren to suppress the *Idua-W402X* nonsense mutation associated with Mucopolysaccharidosis I-Hurler (MPS I-H), a lysosomal storage disease caused by a deficiency of α-L-iduronidase that leads to an accumulation of glycosaminoglycans (GAGs). We found that ataluren restored enough α-L-iduronidase activity in mouse embryonic fibroblasts (MEFs) derived from homozygous *Idua-W402X* mice to reduce GAG storage. Short-term (2-week) oral administration of ataluren to *Idua-W402X* mice also resulted in significant reductions in GAG accumulation within multiple tissues. Together, these results provide important new insights supporting the potential of ataluren as a readthrough agent.

## Materials and methods

### Readthrough drugs

Ataluren for this study was provided by PTC Therapeutics, Incorporated. Amlexanox was purchased from LGM Pharma. Gentamicin and G418 aminoglycosides were purchased from VetOne and Life Technologies (10,131–035), respectively. For all in vitro assays, all compounds other than the aminoglycosides were dissolved in dimethylsulfoxide (DMSO) (Sigma D2650) to a final DMSO concentration of 0.3% (vol/vol). The aminoglycosides were administered in a PBS vehicle.

### Tissue culture

The generation of an immortalized mouse embryonic fibroblast (MEF) cell line from homozygous *Idua-W402X* mice (B6.129S-*Idua*^*tm1.1Kmke*^/J) was previously described [20, 21]. MEF and HEK293 cell lines were cultured at 37 °C with 5% CO_2_ in Dulbecco’s Modification of Eagle’s Medium containing 4.5 g/l glucose, L-glutamine and sodium pyruvate (Corning Cellgro 10–013-CV). This media was supplemented with MEM non-essential amino acids (Corning Cellgro 25–025-Cl) at a final concentration of 1% (v/v) and fetal bovine sera (Atlanta Biologicals S11150) at a final concentration of 10% (v/v). Fisher rat thyroid (FRT) cells were cultured in Nutrient Mixture F-12 Coon’s modification media (Sigma F6636) supplemented with 5% fetal bovine sera. In the absence of stable transformant selection, 100 units/ml penicillin/streptomycin (Corning Cellgro 30–002-Cl) was added to the media to prevent bacterial contamination.

### Construction of NanoLuc readthrough reporters

The pFN[Nluc/CMV/Neo] plasmid containing the NanoLuc open reading frame was purchased from Promega (CS181701). Point mutations were introduced into NanoLuc at codon 12 using site-directed mutagenesis, which changed the tryptophan codon (UGG) to a UGA premature termination codon. At codon Q44, the glutamine codon (CAA) was changed to a UAA stop codon, and at codon K91, the lysine codon (AAG) was changed to a UAG stop codon. The following primers were used to introduce the NanoLuc mutations: W12X: DB4084 (5′-CGT TGG GGA CTG ACG ACA GAC AGC C-3′ and DB4085 (5′-GGC TGT CTG TCG TCA GTC CCC AAC G-3′).; Q44X: DB4175 (5′- CCG TAA CTC CGA TCT AAA GGA TTG TCC TG-3′ and DB4176 (5′-CAG GAC AAT CCT TTA GAT CGG AGT TAC GG-3′); K91X: DB4150 (5′-CAT CAC TTT TAG GTG ATC CTG CAC-3′) and DB4151 (5′-GTG CAG GAT CAC CTA AAA GTG ATG-3′). The resulting PCR reaction was incubated with Dpn I to digest the template DNA and the digest was subsequently transfected into XL1 Blue Supercompetent Cells (Stratagene 200249). Transformants were sequenced to verify the presence of each mutation and to ensure that no additional changes were introduced into the NanoLuc sequence. To stably express the NanoLuc constructs in HEK293 and FRT cells, the wildtype and mutant NanoLuc constructs were each subcloned into the NheI and XhoI sites of pcDNA3.1Zeo(-) (Invitrogen V86520). The plasmid constructs were subsequently transfected into HEK293 and FRT cells with stable transfectants selected using 0.8 mg/ml of zeocin and maintained using 0.2 mg/ml of zeocin (Invitrogen R-250–05).

### NanoLuc activity assay

Prior to performing NanoLuc assays, zeocin was omitted from the media of the stable NanoLuc HEK293 and FRT cell lines for 2 passages due to its inhibitory effect on cell growth. WT and nonsense reporter cells were then seeded into 96-well plates at a density of 4 × 10^4^ cells per well for HEK293 cells and 2 × 10^4^ cells per well for FRTs. Drugs were added when cells became 50% confluent and incubated 48 h prior to assay. NanoLuc activity was measured using the Nano-Glo Luciferase Assay (Promega, N1110). All cells expressing the NanoLuc constructs were lysed in 50 μl of 1X Passive Lysis Buffer (PLB) (Promega, E1941). However, the lysate of WT NanoLuc expressing cells was subsequently diluted 1:1000 with 1X PLB prior to assay. In a separate 96-well plate (Fisher 12–566-04), 5 μl of the Nano-Glo Reagent was mixed with 5 μl of each cell lysate and then incubated for 10 min at room temperature. Luciferase activity readings were then measured using a GloMax (Promega). The data is expressed as the NanoLuc activity generated normalized to micrograms of total protein.

### MEF α-L-iduronidase activity assay

*Idua-W402X* and wild-type MEFs were seeded into 6-well culture dishes at a density of 5 × 10^4^ cells per well. MEFs were grown to 50% confluency and then treated with readthrough agents for 48 h. MEFs were subsequently washed with PBS and lysed in Mammalian Protein Extraction Reagent (Pierce P178501) containing a protease inhibitor cocktail (Roche 11,873,580,001). The total protein concentration was determined using the Bio-Rad Protein Assay (Bio-Rad 5,000,006). Fifty to eighty micrograms of total lysate protein were incubated in a 50 μl reaction containing 0.12 mM 4-methyl-umbelliferyl-α-L-iduronide (Gold Biotech M-570–5; substrate lots used for this study had comparable background fluorescence) and 0.42 mg/ml of D-saccharic acid 1,4-lactone monohydrate (a β-glucuronidase inhibitor) (Sigma S0375) in 130 mM sodium formate buffer, pH 3.5. The reaction was incubated for 48 h at 37 °C and then subsequently quenched with 1 ml of glycine buffer, pH 10.8. The samples were transferred to methacrylate cuvettes and fluorescence at an excitation = 365 nm and an emission = 450 nm was measured using a Cary Eclipse Spectrofluorometer. Free acid 4-methylumbelliferone (FMU) (Sigma M1381) in glycine buffer was used to generate a standard curve. Specific activity was calculated as picomoles of FMU released per milligram of protein per hour. α-L-iduronidase activity remained linear over the 48-h incubation time.

### MEF GAG assay

*Idua-W402X* and wild-type MEFs were incubated with ataluren for 48 h and then lysed using M-Per Protein Reagent (Pierce). GAG levels were determined using the Blyscan Sulfated GAG Assay (Biocolor Ltd, UK CLRB1500). Briefly, 50 µL of each lysate was mixed with 500 µL of the Blyscan Dye Reagent to bind sulfated GAGs. The dye-bound GAGs were pelleted by microfuging for 10 min at 10,000 g at room temperature. Five hundred microliters of the Blyscan Dye Dissociation Reagent was added to each sample to dissociate the GAGs from the dye. The entire volume of each sample was then placed into a cuvette and the absorbance was measured at a wavelength of 656 nm using a Beckman Coulter DU-530 spectrophotometer. The total amount of sulfated GAGs precipitated from each sample was determined from a chondroitin 4-sulfate (Sigma C9819) standard curve. The total protein concentration in each lysate was determined using the Bio-Rad protein assay (Bio-Rad 5,000,006) from a standard curve generated using bovine serum albumin. The data are expressed as nanograms of GAG per milligram of total protein.

### Animal treatment

Ataluren was administered to homozygous wild-type (+ / +) and *Idua-W402X* (− / −) mice. The *Idua-W402X* mice (B6.129S-*Idua*^*tm1.1Kmke*^/J) are available from Jackson Laboratories. Whenever possible, wild-type (+ / +) littermates (obtained from breeding heterozygous (+ / −) *Idua-W402X* mice) were used as controls. Ataluren was administered orally in infused mouse chow (Harlan 7013). Alternatively, ataluren was administered in unflavored Peptamen Liquid Diet (Nestle 6269), which replaced both food and water. Ataluren administration was initiated in 10-week-old male and female mice and continued for 2 weeks. At the end of treatment, animals were perfused with cold PBS and tissues were harvested, flash frozen, and stored at − 80 °C until assayed. All animal work was conducted according to relevant national and international guidelines and all animal protocols used in this study were reviewed and approved by the UAB IACUC (Protocol numbers: APN#120,109,344 and IACUC-10220).

### Tissue GAG assays

This assay was performed as previously described [21, 22]. Tissues were homogenized using a Tissue Tearor homogenizer in chloroform:methanol (2:1 v/v). Defatted tissue was dried in a speedvac and then suspended in 100 mM dibasic sodium phosphate, pH 6.5 containing 0.6 mg/ml cysteine and 2 mg/ml papain (Sigma P4762). The mixture was digested at 60 °C for 18–24 h with constant agitation. The samples were then microfuged at 10,000 g for 15 min and the supernatant was used to quantitate the tissue GAGs using the Blyscan Sulfated GAG Assay (Biocolor Ltd, UK CLRB1500). The total amount of sulfated GAGs precipitated from each sample was determined from a standard curve using chondroitin 4-sulfate (Sigma C9819). The data is expressed as the micrograms of GAGs per milligram of defatted, dried tissue.

## Results

### Ataluren suppresses translation termination at different PTCs in NanoLuc readthrough reporters

**Fig. 1:**
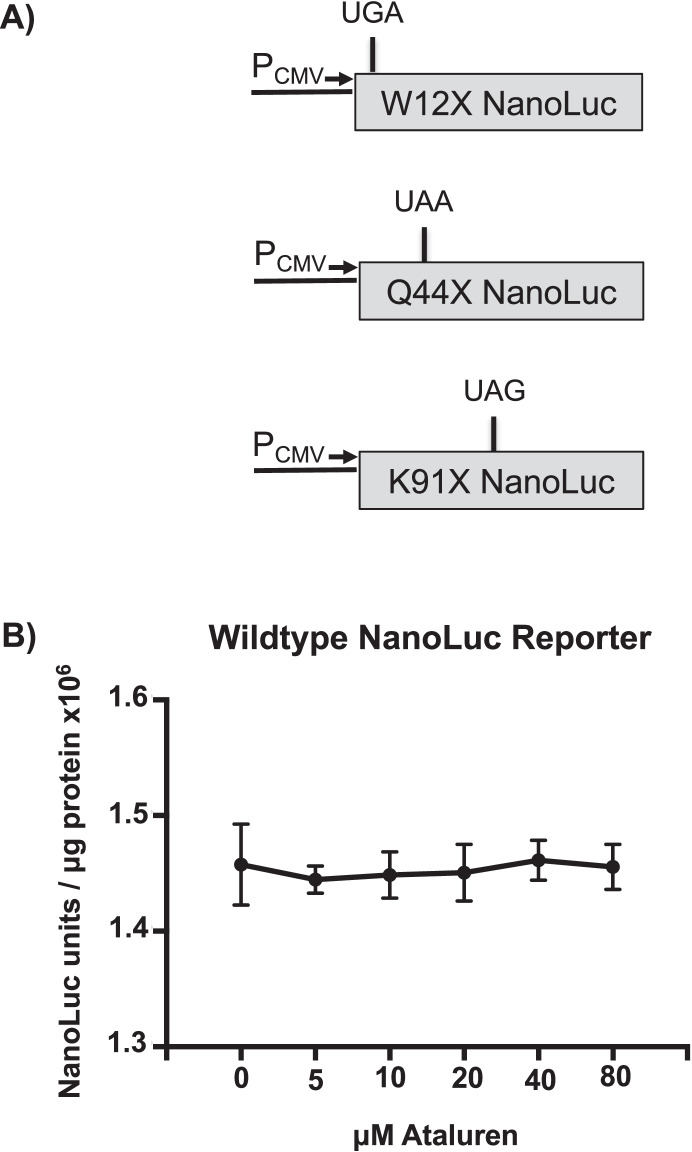
NanoLuc readthrough reporter constructs. Reporter constructs containing PTCs at three different positions within the NanoLuc ORF were used to assess readthrough efficiency of different compounds. **A** NanoLuc readthrough reporters were generated that contain: a UGA at position W12 (W12X); a UAA at position Q44 (Q44X); or a UAG at position K91 (K91X). **B** FRT cells stably expressing a wild-type NanoLuc construct were treated with ataluren at the indicated concentrations for 48 h. NanoLuc activity was then measured in cell lysates. The data is expressed as the mean NanoLuc activity normalized to total protein of two independent experiments, each performed with six replicates

Many studies have shown that ataluren can suppress disease-associated nonsense mutations and restore partial protein function within various in vitro and in vivo systems [5]. However, the ability of ataluren to suppress PTCs has not been without controversy. Because ataluren was previously shown to stabilize firefly luciferase under certain experimental conditions [23–26], we generated new NanoLuc-based reporters to assess the effectiveness of ataluren and other compounds to suppress PTCs in cultured cell systems (Fig. 1A). NanoLuc is an engineered luciferase derived from the deep-sea shrimp *Oplophorus gracilirostris* [27]. It possesses no sequence homology to, and is structurally distinct from, firefly or other known luciferases. Furthermore, the NanoLuc substrate, furimazine, is dissimilar to the D-luciferin substrate required for firefly luciferase. Given these differences in structure and substrate specificity between the NanoLuc and firefly luciferases, it is highly unlikely that ataluren would bind and stabilize NanoLuc luciferase as previously suggested with firefly luciferase [23, 24, 26, 28]. In support of this supposition, no change in NanoLuc activity was observed in HEK293 cells expressing a wild-type NanoLuc control in the presence of ataluren relative to vehicle-treated controls (Fig. 1B).

By changing a single nucleotide at three different positions, different PTCs were introduced into the NanoLuc open reading frame: a UGA at codon W12, a UAA at Q44, and a UAG at K91 (Fig. 1A). These readthrough reporters were stably expressed in two different cells lines: HEK293 cells, which were previously used to evaluate ataluren [14, 19], and Fischer rat thyroid (FRT) cells, which have shown to be permissive to readthrough [29]. In addition to ataluren, both cell types were also treated with two other clinically relevant drugs previously identified as readthrough agents: the aminoglycoside gentamicin [30–32] and amlexanox, an anti-inflammatory compound [33]. For comparison, these cells were treated with G418, a potent but toxic aminoglycoside readthrough agent [11, 34].

**Fig. 2:**
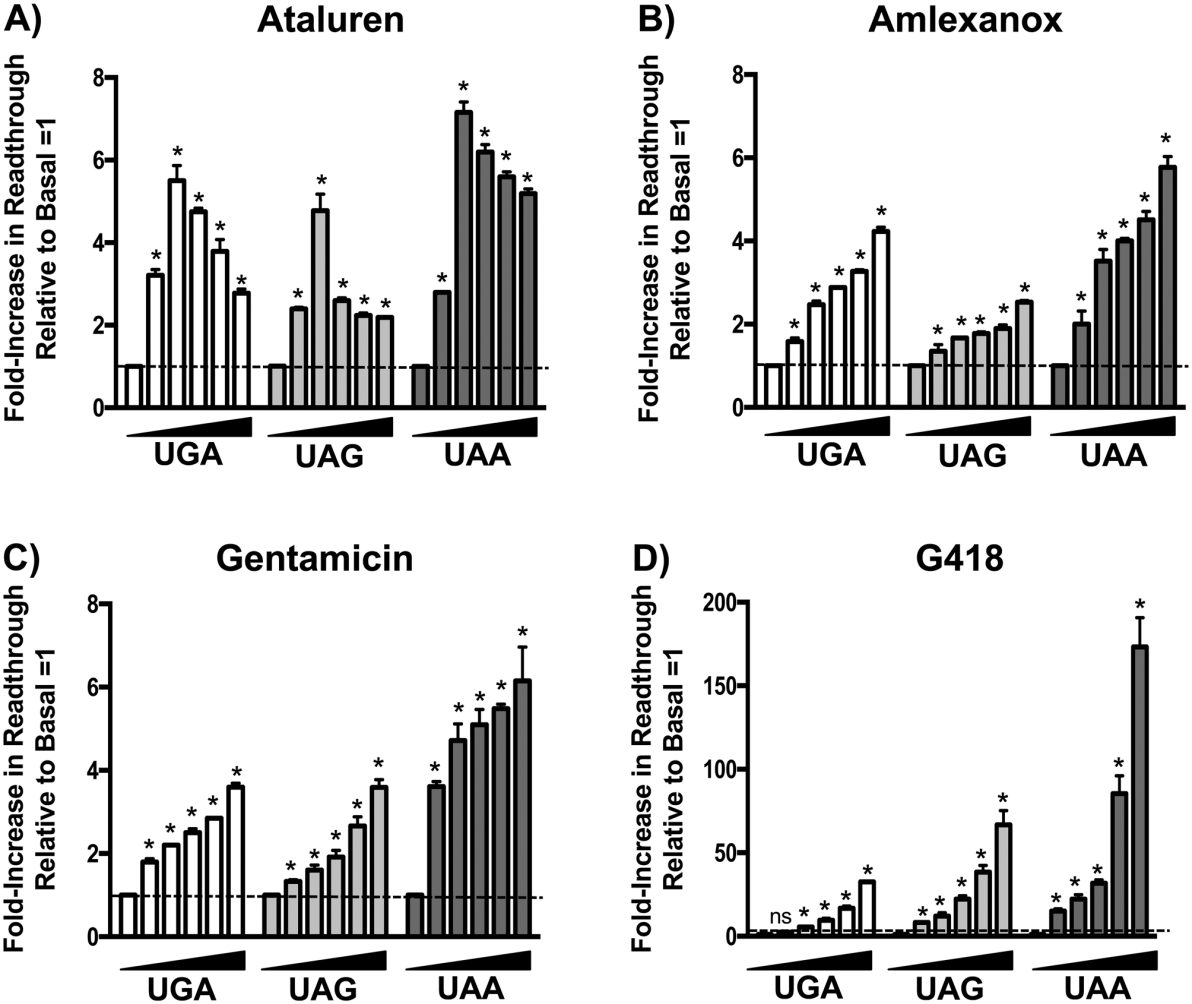
The effect of ataluren and other readthrough agents on NanoLuc readthrough reporters in HEK293 cells. Four different readthrough compounds were evaluated using either the UGA, UAA, or UAG NanoLuc reporters in HEK293 cells. **A** Ataluren, **B** amlexanox, **C** gentamicin, and **D** G418 were assessed. Each compound was examined at concentrations ranging from 5 to 80 μM using twofold concentration steps. Each bar represents the mean ± SD of two independent experiments, each performed in quadruplicate. Exact *p* values were calculated using the unpaired, two-tailed *t*-test comparing the readthrough level in treated cells compared to the vehicle alone controls. * indicates *p* < 0.0001 unless otherwise indicated; *p* > 0.05 = not significant (ns)

**Fig. 3:**
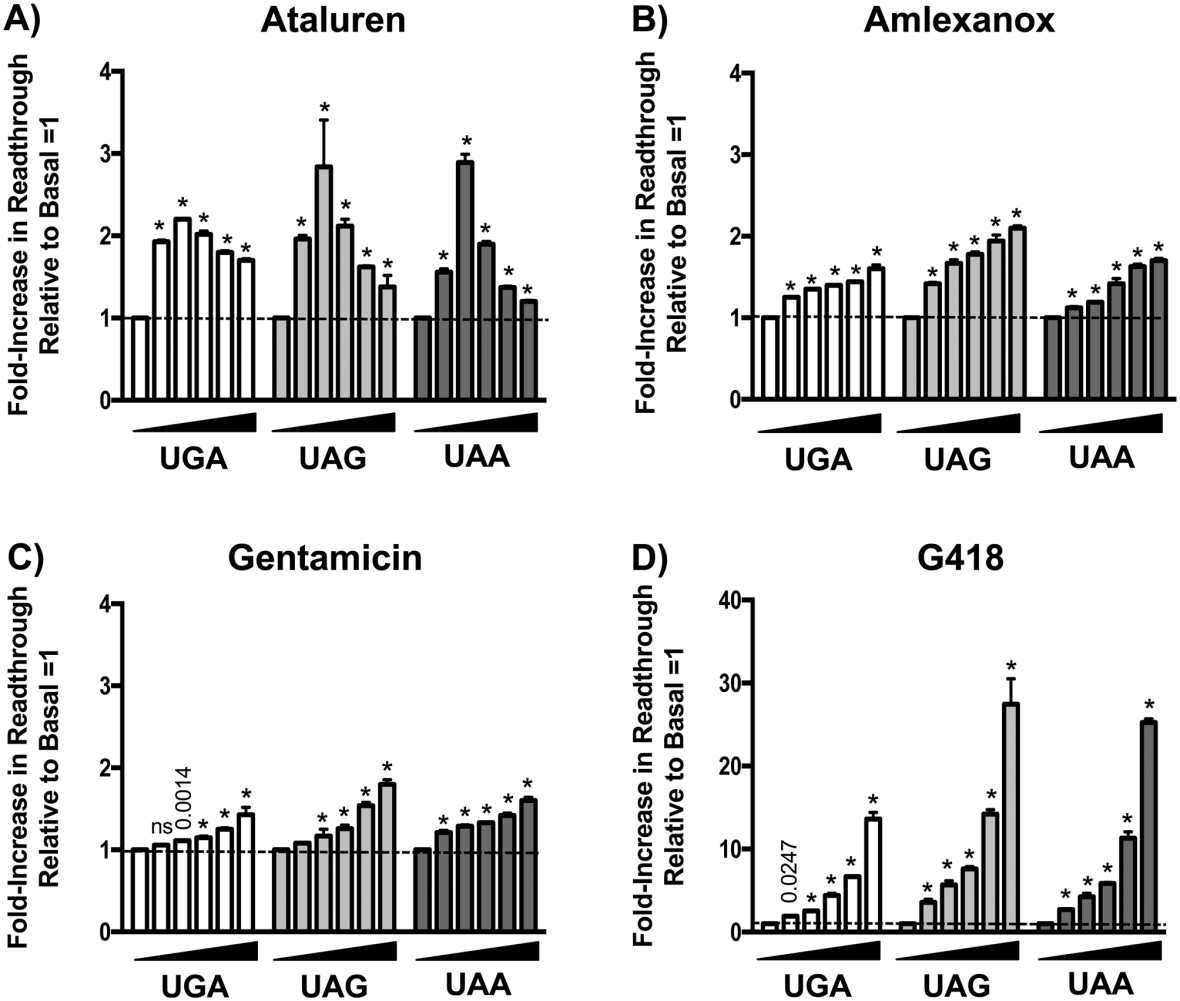
The effect of ataluren and other readthrough agents on NanoLuc readthrough reporters in Fischer rat thyroid (FRT) cells. Four different readthrough compounds were evaluated using either the UGA, UAA, or UAG NanoLuc reporters in FRT cells. They include: **A** ataluren, **B** amlexanox, **C** gentamicin, and **D** G418. Each drug was examined at concentrations ranging from 5 to 80 μM using twofold concentration steps. Each bar represents the mean ± SD of two independent experiments, each performed in quadruplicate. Exact *p* values were calculated using the unpaired, two-tailed *t*-test comparing the readthrough level in treated cells compared to vehicle alone controls. * indicates *p* < 0.0001 unless otherwise indicated; *p* > 0.05 = not significant (ns)

We found that these compounds induced readthrough of the three NanoLuc reporter PTCs in both HEK293 (Fig. 2) and FRT cells (Fig. 3) to varying extents. In both cell types, G418 was the most potent readthrough drug, with NanoLuc activity increasing in a stop codon-dependent manner to a maximum 35- to 170-fold in HEK293 cells (Fig. 2D) and 12- to 28-fold in FRTs (Fig. 3D) relative to vehicle-treated cells. Ataluren was the next most efficient readthrough drug, maximally increasing NanoLuc activity relative to controls from five- to seven-fold in HEK293s (Fig. 2A) and two- to three-fold in FRTs (Fig. 3A). Gentamicin and amlexanox similarly suppressed PTCs relative to basal conditions by a maximum two- to six-fold in HEK293s (Fig. 2B–C), but generally less than two-fold in FRTs (Fig. 3B–C). Notably, G418, gentamicin, and amlexanox all generated a linear dose-dependent curve with the maximum response obtained at 80 μM (the highest dose tested). In contrast, ataluren produced a bell-shaped dose curve in both cell lines, with a 10 μM dose providing maximal readthrough.

Based upon these results, the following general observations could be made concerning the readthrough efficiency of the different drugs tested for each PTC. In HEK293 cells treated with ataluren, amlexanox, or gentamicin, the UAA (Q44X) PTC responded most robustly (as indicated by the fold-increase in readthrough relative to vehicle-treated cells), followed by the UGA (W12X) and UAG (K91X) PTCs (Fig. 2). In FRT cells treated with ataluren (Fig. 3A) or G418 (Fig. 3D), the pattern of stop codon readthrough was UAA = UAG > UGA. However, only minor differences were detected in readthrough among the different PTCs in FRTs treated with amlexanox or gentamicin (Fig. 3B–C). When taken together, these results confirm previous studies that showed ataluren is a bona fide readthrough agent [5]. Furthermore, ataluren induced readthrough of UAA, UAG, and UGA PTCs better than gentamicin or amlexanox in the reporters used here.

### Ataluren suppresses the Idua-W402X nonsense mutation associated with Mucopolysaccharidosis I-Hurler (MPS I-H)

We next tested the ability of ataluren to suppress a PTC associated with the disease MPS I-H. MPS I-H is an autosomal recessive, lysosomal storage disease caused by mutations in the *IDUA* gene that leads to a severe deficiency of α-L-iduronidase, an enzyme that participates in the breakdown of the glycosaminoglycans (GAGs) dermatan sulfate and heparan sulfate. Loss of α-L-iduronidase leads to the accumulation of these GAGs, and subsequently, to the progressive onset of neurological abnormalities and defects in the bone, heart, liver, and spleen, as well as a reduced lifespan. We previously generated a knock-in mouse that carries a single nucleotide change in exon 9 of the mouse *Idua* gene, generating a PTC homologous to the *IDUA-W402X* nonsense mutation found in MPS I-H patients [35]. Homozygous *Idua-W402X* mice have a severe deficiency of α-L-iduronidase, resulting in the onset of phenotypes that closely recapitulate disease progression in MPS I-H patients [20–22, 35]. We previously used this model to show that the non-traditional, designer aminoglycoside, NB84, can suppress the *Idua-W402X* mutation and restore enough α-L-iduronidase activity to reduce GAG accumulation in both short-term [21, 22] and long-term studies [21, 22]. We therefore used this well-characterized model to examine whether ataluren is also capable of suppressing the *Idua-W402X* nonsense mutation.

**Fig. 4:**
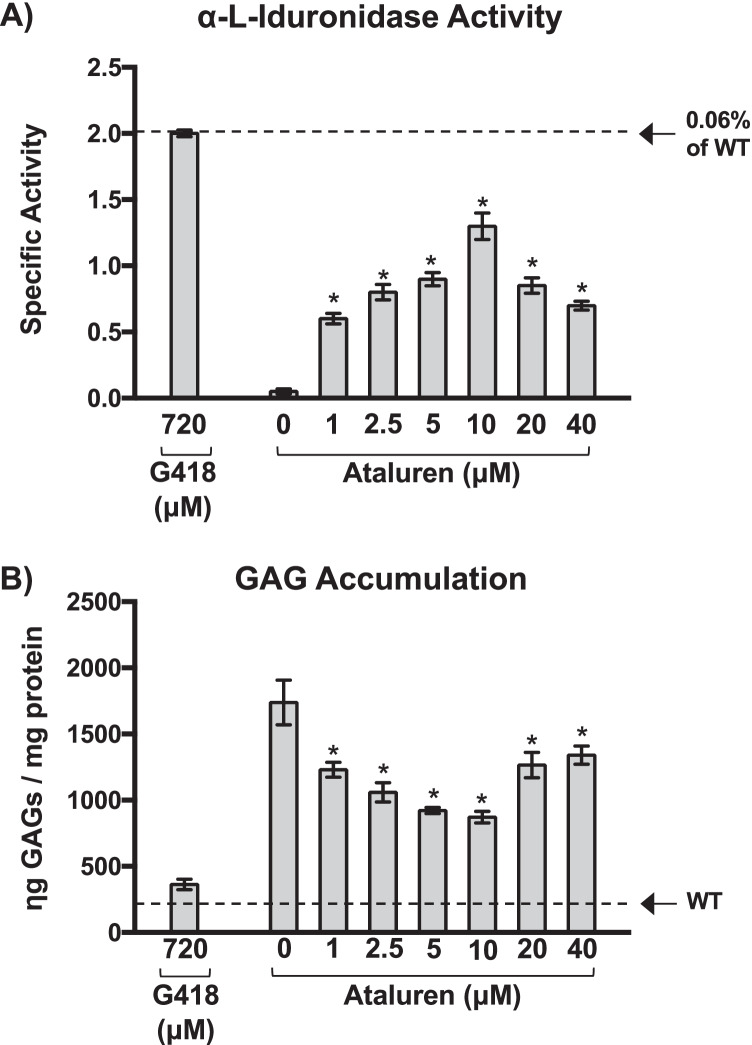
Ataluren restores enough α-L-iduronidase activity to reduce GAG accumulation in *Idua-W402X* mouse embryonic fibroblasts (MEFs). Immortalized MEFs derived from homozygous *Idua-W402X* mice were cultured in the presence of G418 or ataluren for 48 h. MEF lysates were then generated to measure: **A** α-L-iduronidase specific activity (picomoles of FMU released per milligram of protein per hour), or **B** sulfated GAG storage. Each bar represents the mean ± SD of a representative experiment (*n* = 6). Exact *p* values were calculated using the unpaired, two-tailed *t*-test comparing the values in treated cells compared to vehicle-treated controls. * indicates *p* < 0.0001 unless otherwise indicated. *p* < 0.0001 when comparing all mutant and wild-type samples

We first examined the ability of ataluren to restore α-L-iduronidase in immortalized mouse embryonic fibroblasts (MEFs) derived from homozygous *Idua*-*W402X* mice. α-L-iduronidase specific activity was determined in MEF lysates using a fluorescent substrate as previously described [20–22]. Compared to vehicle-treated controls, we found a maximum ten-fold increase in α-L-iduronidase activity in *Idua-W402X* MEFs cultured with ataluren for 48 h that corresponded to approximately 0.045% of wild-type activity (Fig. 4A). To determine whether this increase in α-L-iduronidase activity was sufficient to reduce GAG accumulation, we quantitated sulfated GAG levels using a GAG dye-binding assay as previously described [20–22]. We found that GAGs were reduced by as much as 63% in *Idua-W402X* MEFs treated with ataluren compared to the vehicle control (Fig. 4B). This suggests that the level of α-L-iduronidase activity restored by ataluren-mediated readthrough of the *Idua-W402X* mutation was sufficient to moderate the primary biochemical defect associated with MPS I-H. Consistent with our NanoLuc readthrough reporter results in HEK293 and FRT cells, we observed that ataluren also exhibited a bell-shaped dose response for the α-L-iduronidase and GAG assays in MEFs, where a 10 μM dose produced the maximum response for both assays.

**Fig. 5:**
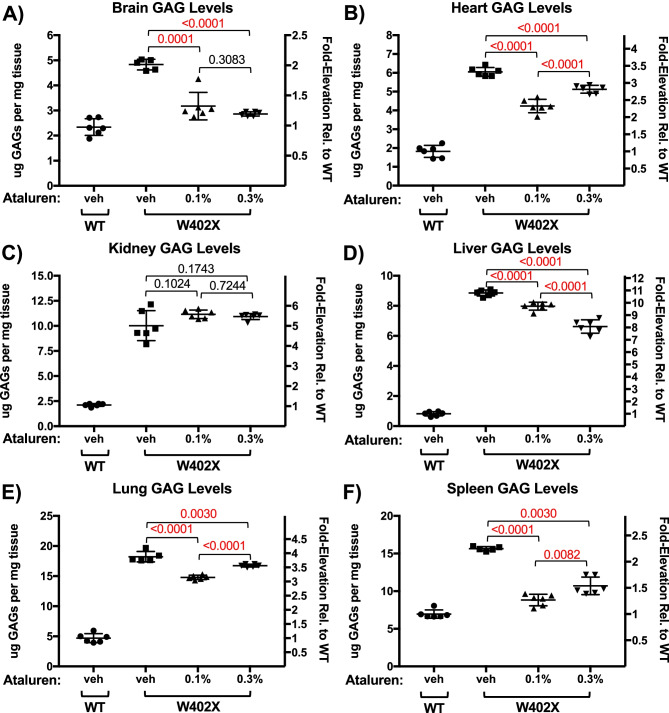
Ataluren significantly reduces GAG accumulation within most tissues from *Idua-W402X* mice when administered orally. Five-week-old *Idua-W402X* mice were orally administered ataluren-infused mouse chow at the indicated dose (% = mass/mass) for 2 weeks. Sulfated GAG levels were then quantified in the following tissues: **A** brain, **B** heart, **C** kidney, **D** liver, **E** lung, and **F** spleen. Each data point represents an average assay value (performed in quadruplicate) from a single mouse. Bars indicate the group mean ± SD. Exact *p* values were calculated using the unpaired, two-tailed *t*-test to compare the bracketed cohorts. *p* < 0.005 when comparing all wild-type and W402X cohorts. *n* = 5–6 mice per cohort

**Fig. 6:**
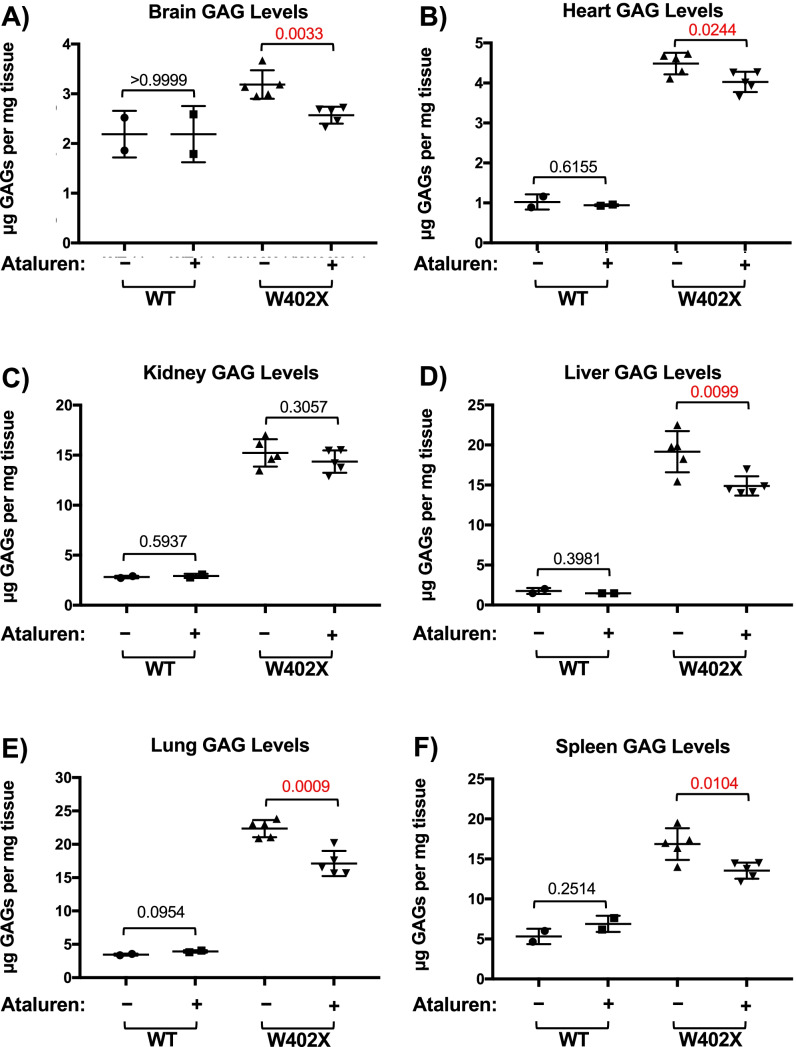
Ataluren administration using a previously published dosing regimen also significantly reduces GAG accumulation within most *Idua-W402X* mouse tissues. 8- to 9-week-old *Idua-W402X* mice were orally administered 0.9 mg/ml ataluren in Peptamen liquid diet for 2 weeks. Sulfated GAG levels were then quantified in the following mouse tissues: **A** brain, **B** heart, **C** kidney, **D** liver, **E** lung, **F** spleen. Each data point represents an average assay value (performed in quadruplicate) from a single mouse. Bars indicate the group mean ± sd. Exact *p* values were calculated using the unpaired, two-tailed, *t*-test for the bracketed cohorts. *p* < 0.0001 when comparing all wild-type and W402X cohorts. *n* = 2–6 mice per cohort

We next evaluated whether ataluren could suppress the *Idua-W402X* nonsense mutation in vivo. *Idua-W402X* mice were treated with different concentrations of ataluren blended with mouse chow and administered ad libitum for 2 weeks. After treatment, we attempted to monitor α-L-iduronidase activity in tissue lysates, but significant quenching of fluorescence by tissue lysate components precluded accurate detection of enzyme activity in mutant mouse tissues. However, we were able to measure the restoration of α-L-iduronidase activity indirectly by quantitating sulfated GAG levels in mouse tissue lysates using a GAG dye-binding assay as previously described [20–22] (Fig. 5). Compared to vehicle alone controls, we found a 30–50% reduction in GAG storage among all the tissues assayed from ataluren-treated *Idua-W402X* mice, except for the kidney, which showed no significant change in GAG levels. Notably, we also found a bell-shaped ataluren dose response for the GAG assay in MPS I-H mice in three tissues examined (heart, lung, and spleen). The 0.3% ataluren dose was most effective at reducing GAGs in the liver, while the responses to both doses were similar in the brain. We also administered ataluren to *Idua-W402X* and wild-type mice for 2 weeks in a liquid diet at a 0.9 mg/ml dose, which is comparable to the 0.1% chow dose and was previously found to suppress nonsense mutations in a mouse model of cystic fibrosis [36] (Fig. 6). We found that GAG levels remained unchanged in wild-type mice treated with ataluren relative to vehicle alone controls. However, in *Idua-W402X* mice treated with ataluren, we found significant reductions in GAG levels among most tissues examined, ranging from modest 10% reductions in the heart and liver, to more robust 30–60% reductions in the brain, lung, and spleen. Once again, a significant reduction was not observed in the kidneys. Overall, these data suggest that ataluren can suppress the *Idua-W402X* nonsense mutation at levels sufficient to reduce GAG accumulation in most, but not all tissues.

## Discussion

The ability of ataluren to suppress PTCs has been controversial based mainly upon three findings. First, under certain experimental conditions, ataluren can bind and stabilize firefly luciferase [23, 24, 26, 28], which was the reporter used to initially identify ataluren [14]. This led to the suggestion that its identification may have been an artifact. A subsequent report discounted this possibility under the experimental conditions that were used to identify ataluren [28]. Second, the function of ataluren as a readthrough agent has also been challenged by two studies in which it was reported that ataluren was unable to suppress PTCs within multiple in vitro reporters [19, 37]. Finally, results from randomized, double-blinded, placebo-controlled phase 3 clinical trials in which ataluren was administered to cystic fibrosis patients harboring PTCs showed no significant improvements in lung function [18].

However, there is also an abundance of data demonstrating that ataluren functions as a readthrough drug. It has been demonstrated that PTC suppression mediated by ataluren can restore the function of many proteins that are structurally unrelated to each other or to firefly luciferase [5]. Proteins whose expression and/or function have been restored by ataluren treatment include CFTR [36], dystrophin [14], harmonin [38], α-L-iduronidase (this study), and many others (see Peltz et al. for a review) [5]. The diversity among these proteins suggests that it is highly unlikely that ataluren increases the function of these proteins through an ability to bind and stabilize full-length proteins that arise from basal readthrough. Furthermore, direct evidence that ataluren mediates readthrough in mammalian cells was obtained using mass spectrometry to show that ataluren promotes the insertion of aminoacyl-tRNAs at PTCs [17].

In the current study, we found that ataluren promotes PTC suppression in both HEK293 and FRT cells, as demonstrated by an increase in activity for all three NanoLuc reporters (Figs. 2 and 3). While the level of readthrough achieved with ataluren was not as robust as that observed with G418, ataluren was more effective at promoting readthrough than either gentamicin or amlexanox. A notable difference between ataluren and the other readthrough drugs examined is the dose–response curve. G418, gentamicin, and amlexanox demonstrated an S-shaped dose response with NanoLuc readthrough assays in both HEK293 and FRT cells. However, ataluren resulted in a bell-shaped dose response with not only the NanoLuc readthrough assays, but also the α-L-iduronidase and GAG assays in MEFs and the tissue GAG quantitation from ataluren-treated *Idua-W402X* mice. It was previously shown that the aminoglycoside tobramycin inhibits readthrough by ataluren, suggesting that like the aminoglycosides, ataluren also likely binds to the ribosome to induce readthrough [17]. While aminoglycosides (such as G418 and gentamicin) bind specifically to one region of the 18S ribosomal RNA known as the decoding site to induce readthrough at PTCs [39], the bell-shaped dose response of ataluren suggests that it may have multiple binding sites with different binding affinities. More recent studies have shown that G418 stimulates readthrough by near-cognate mispairing while ataluren promotes readthrough by inhibiting release factor activity [40]. We speculate that ataluren may bind to a higher affinity site to induce readthrough, while binding to a lower affinity site abrogates its readthrough activity. This unusual pharmacokinetic profile also suggests that ataluren may have a narrow therapeutic window. We propose that the atypical dose–response profile of ataluren relative to other readthrough drugs is likely to be a major factor contributing to the inability of some studies to demonstrate ataluren-mediated readthrough.

We also examined the effect of ataluren on the suppression of the *Idua*-*W402X* genomic nonsense mutation. We found that ataluren suppressed the *Idua-W402X* nonsense mutation in MEFs, as demonstrated by an increase in α-L-iduronidase activity and a corresponding 60% decrease in GAGs relative to vehicle controls (Fig. 4A). Importantly, this reduction results in GAG levels previously reported to be associated with an attenuated MPS I phenotype [41]. Oral administration of ataluren to *Idua-W402X* mice for 2 weeks also resulted in a significant GAG reduction within multiple tissues (Figs. 5 and 6). This level of GAG reduction was previously reported to attenuate MPS I-H progression in multiple tissues of *Idua-W402X* mice [22] and correspond to GAG levels observed with an attenuated MPS I phenotype in patients [41]. Consistent with the wide tissue distribution of ataluren [15, 16], GAGs were significantly reduced in the brain and heart, tissues that are recalcitrant to current MPS I-H treatments including hematopoietic stem cell transplantation and enzyme replacement therapy. Importantly, wild-type mice administered ataluren showed no difference in tissue GAG levels relative to vehicle controls (Fig. 6), demonstrating the specificity of ataluren readthrough action.

In clinical trials, ataluren did not significantly improve lung disease in cystic fibrosis patients, for whom at least 30–35% of normal CFTR function is needed to alleviate pulmonary dysfunction [42]. However, ataluren may be effective for other genetic diseases that have a lower threshold for correction. For example, as little as ~ 0.3% of wildtype α-L-iduronidase activity can significantly attenuate clinical symptoms in MPS I-H patients [43]. Notably, ataluren has been approved by the European Medicines Agency for treatment of Duchenne muscular dystrophy (DMD) patients who carry nonsense mutations and additional DMD clinical trials are currently underway in the USA (ClinicalTrials.gov Identifiers: NCT04336826 & NCT03179631). Additional clinical studies will be required to determine whether ataluren may be an effective readthrough agent for MPS I-H or other genetic diseases that result from PTCs.

## Data Availability

All relevant data generated and/or analyzed during the current study are shown in the article.

## References

[1] Keeling KM, Xue X, Gunn G, Bedwell DM (2014). Therapeutics based on stop codon readthrough. Annu Rev Genomics Hum Genet.

[2] Zhouravleva G, Frolova L, Le Goff X, Le Guellec R, Inge-Vechtomov S, Kisselev L, Philippe M (1995). Termination of translation in eukaryotes is governed by two interacting polypeptide chain release factors, eRF1 and eRF3. EMBO J.

[3] Fearon K, McClendon V, Bonetti B, Bedwell DM (1994). Premature translation termination mutations are efficiently suppressed in a highly conserved region of yeast Ste6p, a member of the ATP-binding cassette (ABC) transporter family. J Biol Chem.

[4] Lee HL, Dougherty JP (2012). Pharmaceutical therapies to recode nonsense mutations in inherited diseases. Pharmacol Ther.

[5] Peltz SW, Morsy M, Welch EM, Jacobson A (2013). Ataluren as an agent for therapeutic nonsense suppression. Annu Rev Med.

[6] Hutchin T, Cortopassi G (1994). Proposed molecular and cellular mechanism for aminoglycoside ototoxicity. Antimicrob Agents Chemother.

[7] Quiros Y, Vicente-Vicente L, Morales AI, Lopez-Novoa JM, Lopez-Hernandez FJ (2011). An integrative overview on the mechanisms underlying the renal tubular cytotoxicity of gentamicin. Toxicol Sci.

[8] Mingeot-Leclercq MP, Tulkens PM (1999). Aminoglycosides: nephrotoxicity. Antimicrob Agents Chemother.

[9] Shulman E, Belakhov V, Wei G, Kendall A, Meyron-Holtz EG, Ben-Shachar D, Schacht J, Baasov T (2014). Designer aminoglycosides that selectively inhibit cytoplasmic rather than mitochondrial ribosomes show decreased ototoxicity: a strategy for the treatment of genetic diseASES. J Biol Chem.

[10] Dagil R, O'Shea C, Nykjaer A, Bonvin AM, Kragelund BB (2013). Gentamicin binds to the megalin receptor as a competitive inhibitor using the common ligand binding motif of complement type repeats: insight from the NMR structure of the 10th complement type repeat domain alone and in complex with gentamicIN. J Biol Chem.

[11] Kandasamy J, Atia-Glikin D, Shulman E, Shapira K, Shavit M, Belakhov V, Baasov T (2012). Increased selectivity toward cytoplasmic versus mitochondrial ribosome confers improved efficiency of synthetic aminoglycosides in fixing damaged genes: a strategy for treatment of genetic diseases caused by nonsense mutations. J Med Chem.

[12] Guthrie OW (2008). Aminoglycoside induced ototoxicity. Toxicology.

[13] Mingeot-Leclercq MP, Piret J, Brasseur R, Tulkens PM (1990). Effect of acidic phospholipids on the activity of lysosomal phospholipases and on their inhibition by aminoglycoside antibiotics–I. Biochem Anal Biochem Pharmacol.

[14] Welch EM, Barton ER, Zhuo J, Tomizawa Y, Friesen WJ, Trifillis P, Paushkin S, Patel M, Trotta CR, Hwang S (2007). PTC124 targets genetic disorders caused by nonsense mutations. Nature.

[15] Hirawat S, Welch EM, Elfring GL, Northcutt VJ, Paushkin S, Hwang S, Leonard EM, Almstead NG, Ju W, Peltz SW (2007). Safety, tolerability, and pharmacokinetics of PTC124, a nonaminoglycoside nonsense mutation suppressor, following single- and multiple-dose administration to healthy male and female adult volunteers. J Clin Pharmacol.

[16] Hirawat S, Northcutt VJ, Welch EM, Elfring GL, Hwang S, Almstead NG, Ju W, Miller LL (2004). Phase 1 safety and PK study of PTC124 for nonsense-mutation suppression therapy of cystic fibrosis. Pediatr Pulmonol.

[17] Roy B, Friesen WJ, Tomizawa Y, Leszyk JD, Zhuo J, Johnson B, Dakka J, Trotta CR, Xue X, Mutyam V (2016). Ataluren stimulates ribosomal selection of near-cognate tRNAs to promote nonsense suppression. Proc Natl Acad Sci U S A.

[18] Kerem E, Konstan MW, De Boeck K, Accurso FJ, Sermet-Gaudelus I, Wilschanski M, Elborn JS, Melotti P, Bronsveld I, Fajac I (2014). Ataluren for the treatment of nonsense-mutation cystic fibrosis: a randomised, double-blind, placebo-controlled phase 3 trial. Lancet Respir Med.

[19] McElroy SP, Nomura T, Torrie LS, Warbrick E, Gartner U, Wood G, McLean WHI (2013). A lack of premature termination codon read-through efficacy of PTC124 (Ataluren) in a diverse array of reporter assays. PLoS Biol.

[20] Keeling KM, Wang D, Dai Y, Murugesan S, Chenna B, Clark J, Belakhov V, Kandasamy J, Velu SE, Baasov T (2013). Attenuation of nonsense-mediated mRNA decay enhances in vivo nonsense suppression. PLoS ONE.

[21] Wang D, Belakhov V, Kandasamy J, Baasov T, Li SC, Li YT, Bedwell DM, Keeling KM (2012). The designer aminoglycoside NB84 significantly reduces glycosaminoglycan accumulation associated with MPS I-H in the Idua-W392X mouse. Mol Genet Metab.

[22] Gunn G, Dai Y, Du M, Belakhov V, Kandasamy J, Schoeb TR, Baasov T, Bedwell DM, Keeling KM (2013). Long-term nonsense suppression therapy moderates MPS I-H disease progression. Mol Genet Metab.

[23] Auld DS, Lovell S, Thorne N, Lea WA, Maloney DJ, Shen M, Rai G, Battaile KP, Thomas CJ, Simeonov A (2010). Molecular basis for the high-affinity binding and stabilization of firefly luciferase by PTC124. Proc Natl Acad Sci U S A.

[24] Auld DS, Thorne N, Maguire WF, Inglese J (2009). Mechanism of PTC124 activity in cell-based luciferase assays of nonsense codon suppression. Proc Natl Acad Sci U S A.

[25] Peltz SW, Welch EM, Trotta CR, Davis T, Jacobson A (2009). Targeting post-transcriptional control for drug discovery. RNA Biol.

[26] Inglese J, Thorne N, Auld DS (2009) Reply to Peltz et al: Post-translational stabilization of the firefly luciferase reporter by PTC124 (Ataluren). Proc Natl Acad Sci U S A 106: E65

[27] Hall MP, Unch J, Binkowski BF, Valley MP, Butler BL, Wood MG, Otto P, Zimmerman K, Vidugiris G, Machleidt T (2012). Engineered luciferase reporter from a deep sea shrimp utilizing a novel imidazopyrazinone substrate. ACS Chem Biol.

[28] Peltz SW, Welch EM, Jacobson A, Trotta CR, Naryshkin N, Sweeney HL, Bedwell DM (2009). Nonsense suppression activity of PTC124 (ataluren). Proc Natl Acad Sci U S A.

[29] Sharma J, Du M, Wong E, Mutyam V, Li Y, Chen J, Wangen J, Thrasher K, Fu L, Peng N (2021). A small molecule that induces translational readthrough of CFTR nonsense mutations by eRF1 depletion. Nat Commun.

[30] Bedwell DM, Kaenjak A, Benos DJ, Bebok Z, Bubien JK, Hong J, Tousson A, Clancy JP, Sorscher EJ (1997). Suppression of a CFTR premature stop mutation in a bronchial epithelial cell line. Nat Med.

[31] Howard M, Frizzell RA, Bedwell DM (1996). Aminoglycoside antibiotics restore CFTR function by overcoming premature stop mutations. Nat Med.

[32] Barton-Davis ER, Cordier L, Shoturma DI, Leland SE, Sweeney HL (1999). Aminoglycoside antibiotics restore dystrophin function to skeletal muscles of mdx mice. J Clin Invest.

[33] Gonzalez-Hilarion S, Beghyn T, Jia J, Debreuck N, Berte G, Mamchaoui K, Mouly V, Gruenert DC, Deprez B, Lejeune F (2012). Rescue of nonsense mutations by amlexanox in human cells. Orphanet J Rare Dis.

[34] Nudelman I, Glikin D, Smolkin B, Hainrichson M, Belakhov V, Baasov T (2010). Repairing faulty genes by aminoglycosides: development of new derivatives of geneticin (G418) with enhanced suppression of diseases-causing nonsense mutations. Bioorg Med Chem.

[35] Wang D, Shukla C, Liu X, Schoeb TR, Clarke LA, Bedwell DM, Keeling KM (2010). Characterization of an MPS I-H knock-in mouse that carries a nonsense mutation analogous to the human IDUA-W402X mutation. Mol Genet Metab.

[36] Du M, Liu X, Welch EM, Hirawat S, Peltz SW, Bedwell DM (2008). PTC124 is an orally bioavailable compound that promotes suppression of the human CFTR-G542X nonsense allele in a CF mouse model. Proc Natl Acad Sci U S A.

[37] Bolze F, Mocek S, Zimmermann A, Klingenspor M (2017). Aminoglycosides, but not PTC124 (Ataluren), rescue nonsense mutations in the leptin receptor and in luciferase reporter genes. Sci Rep.

[38] Goldmann T, Overlack N, Wolfrum U, Nagel-Wolfrum K (2011). PTC124 mediated translational read-through of a nonsense mutation causing Usher type 1C. Hum Gene Ther.

[39] Lynch SR, Puglisi JD (2001). Structure of a eukaryotic decoding region A-site RNA. J Mol Biol.

[40] Ng MY, Li H, Ghelfi MD, Goldman YE, Cooperman BS (2021). Ataluren and aminoglycosides stimulate read-through of nonsense codons by orthogonal mechanisms. Proc Natl Acad Sci U S A.

[41] Bunge S, Clements PR, Byers S, Kleijer WJ, Brooks DA, Hopwood JJ (1998). Genotype-phenotype correlations in mucopolysaccharidosis type I using enzyme kinetics, immunoquantification and *in vitro* turnover studies. Biochim Biophys Acta.

[42] Kerem E (2004). Pharmacologic therapy for stop mutations: how much CFTR activity is enough?. Curr Opin Pulm Med.

[43] Oussoren E, Keulemans J, van Diggelen OP, Oemardien LF, Timmermans RG, van der Ploeg AT, Ruijter GJ (2013). Residual alpha-L-iduronidase activity in fibroblasts of mild to severe Mucopolysaccharidosis type I patients. Mol Genet Metab.

